# Extrahippocampal Contributions to Age-Related Changes in Spatial Navigation Ability

**DOI:** 10.3389/fnhum.2018.00272

**Published:** 2018-07-10

**Authors:** Jimmy Y. Zhong, Scott D. Moffat

**Affiliations:** School of Psychology, Georgia Institute of Technology, Atlanta, GA, United States

**Keywords:** spatial navigation, cognitive aging, associative learning and memory, executive functioning, prefrontal cortex

## Abstract

Age-related decline in spatial navigation is well-known and the extant literature emphasizes the important contributions of a hippocampus-dependent spatial navigation system in mediating this decline. However, navigation is a multifaceted cognitive domain and some aspects of age-related navigational decline may be mediated by extrahippocampal brain regions and/or systems. The current review presents an overview of some key cognitive domains that contribute to the age-related changes in spatial navigation ability, and elucidates such domains in the context of an increased engagement of navigationally relevant extrahippocampal brain regions with advancing age. Specifically, this review focuses on age-related declines in three main areas: (i) allocentric strategy use and switching between egocentric and allocentric strategies, (ii) associative learning of landmarks/locations and heading directions, and (iii) executive functioning and attention. Thus far, there is accumulating neuroimaging evidence supporting the functional relevance of the striatum for egocentric/response strategy use in older adults, and of the prefrontal cortex for mediating executive functions that contribute to successful navigational performance. Notably, the functional role of the prefrontal cortex was particularly emphasized via the proposed relevance of the fronto-locus coeruleus noradrenergic system for strategy switching and of the fronto-hippocampal circuit for landmark-direction associative learning. In view of these putative prefrontal contributions to navigation-related functions, we recommend future spatial navigation studies to adopt a systems-oriented approach that investigates age-related alterations in the interaction between the prefrontal cortex, the hippocampus, and extrahippocampal regions, as well as an individual differences approach that clarifies the differential engagement of prefrontal executive processes among older adults.

## Introduction

Spatial navigation ability is crucial for everyday living, allowing us to be cognizant of our position and orientation in our environment, as well as helping us to maintain a sense of direction when navigating to and from various locations (Wolbers and Hegarty, 2010; Chersi and Burgess, 2015). Even though spatial navigation seems effortless at the behavioral level, it is a multimodal activity that draws upon a multitude of cognitive and neural resources (Moffat, 2009, 2016; Wolbers and Hegarty, 2010; Wolbers, 2015; Zhong and Kozhevnikov, 2016; Lester et al., 2017). Numerous behavioral studies of spatial navigation in the cognitive aging literature have identified age-related declines or deficits in *navigation strategies* (Moffat and Resnick, 2002; Bohbot et al., 2012; Harris et al., 2012; Wiener et al., 2013; Harris and Wolbers, 2014; Colombo et al., 2017; Zhong et al., 2017), *associative learning/memory* (Head and Isom, 2010; Liu et al., 2011; Wiener et al., 2012, 2013; Zhong and Moffat, 2016; Allison and Head, 2017; O’Malley et al., 2018), and *working memory* (Mahmood et al., 2009; Taillade et al., 2013a,b, 2016; Ariel and Moffat, 2018). Complementary neuroimaging studies that investigated age-related declines in spatial navigation performance and memory have largely linked them to age-related reduction in the volume or activation of the hippocampus (e.g., Driscoll et al., 2003, 2005; Astur et al., 2006; Moffat et al., 2006, 2007; Antonova et al., 2009; Yuan et al., 2014; Daugherty et al., 2015, 2016), a region that has long been proposed as the neural basis of a “cognitive map” (O’Keefe and Dostrovsky, 1971; O’Keefe and Nadel, 1978).

Notwithstanding the pertinence of the hippocampus for spatial navigation, the complexities of navigation-related cognition and behavior cannot be traced to one region alone, and the functional relevance of extrahippocampal brain regions should not be minimized (Doeller et al., 2008; Chersi and Burgess, 2015). Navigationally relevant extrahippocampal regions encompass the striatum, the prefrontal cortex, the parietal cortex and the retrosplenial cortex (RSC; see Moffat et al., 2006, 2007; Moffat, 2009, 2016; Wolbers and Hegarty, 2010; Wolbers, 2015; Lester et al., 2017). Moreover, navigation depends on the contributions of cognitive domains/brain systems that are not specifically navigation-related (e.g., working memory, attention, motor control, etc.) and age differences in these cognitive domains may contribute to and manifest as deficits in navigation tasks. With advancing age, spatial learning and navigational performance start to become more dependent on extrahippocampal regions instead of the hippocampus *per se* (Moffat et al., 2006, 2007; Lester et al., 2017). With a focus on extrahippocampal regions and processes, the current review presents an overview of some key cognitive domains of spatial navigation that are adversely affected by normal aging and relates them to changes in the functional integrity of both hippocampal and extrahippocampal regions. To facilitate organization, we surveyed the literature on aging and navigation strategies and associative learning before turning to a discussion of other processes that are generally executive in nature and largely dependent on the prefrontal cortex. We acknowledge the inherently complex nature of spatial navigation and do not intend to suggest that these cognitive processes and the sections herein are mutually exclusive. Table 1 shows a summary of the main cognitive aging studies covered by this review and the cognitive domains and brain systems investigated or discussed.

**Table 1:** Main cognitive aging studies that investigated and/or discussed extrahippocampal cognitive domains in spatial navigation.

Extrahippocampal	Study	Brain region/system	Regional activation:	Regional activation:
cognitive domain		involved or implicated	Old > Young	Young > Old
Egocentric/response-based	Antonova et al. (2009)	Striatum/caudate nucleus	✓ (see Konishi et al., 2013;
navigation strategy use	Bohbot et al. (2012)	Hippocampal-striatal circuitry	Schuck et al., 2015)	
	Harris et al. (2012)			
	Harris and Wolbers (2014)			
	Iaria et al. (2003)			
	Konishi et al. (2013)			
	Konishi and Bohbot (2013)			
	Rodgers et al. (2012)			
	Schuck et al. (2015)			
	Wiener et al. (2012, 2013)			
	Zhong et al. (2017)			
Switching between allocentric and	Harris et al. (2012)	Locus coeruleus	N.A.	N.A.
egocentric/response strategies	Harris and Wolbers (2014)	Fronto-locus coeruleus		
		noradrenergic system		
		Fronto-hippocampal circuitry		
	Moffat et al. (2006)	Retrosplenial cortex		✓ (in BA 29, see
		(BA 29/30)		Moffat et al., 2006)
Associative learning/memory	Allison and Head (2017)	Dorsolateral striatum	N.A.	N.A.
	Head and Isom (2010)	Fronto-hippocampal circuitry		
	Liu et al. (2011)			
	O’Malley et al. (2018)			
	Wiener et al. (2012, 2013)			
	Zhong and Moffat (2016)			
Executive functioning and attention	Ariel and Moffat (2018)	Medial prefrontal cortex	✓ (in BA 10,	
	Dowiasch et al. (2015)	(BA 9/10)	see Moffat et al., 2006)	
	Driscoll et al. (2005)			
	Hartmeyer et al. (2017)			
	Merriman et al. (2018)			
	Moffat and Resnick (2002)			
	Moffat et al. (2006, 2007)			
	Szturm et al. (2017)			
	Taillade et al. (2013a,b, 2016)			

*Note. The listed studies involved both young and older human participants. Studies showing comparatively higher activation in a specific brain region in one age group over the other are indicated by relevant check marks. “N.A.” denotes “not applicable” for the studies of strategy switching and associative learning without published neuroimaging findings*.

## Navigation Strategies and Strategy Switching

A major theme in the discussion of spatial navigation pertains to navigation strategies, which commonly take the form of mental imagery techniques that require the primary engagement of an egocentric (i.e., first-person) or allocentric (i.e., third-person) frame of reference (see, e.g., Zhong and Kozhevnikov, 2016; He and McNamara, 2018; McCunn and Gifford, 2018; see also Colombo et al., 2017; for a review). While an egocentric navigation strategy requires the moving agent to visualize scenes and gauge self-to-object relationships from a body-centered viewpoint, an allocentric navigation strategy requires the agent to visualize scenes and map out object-to-object relationships from a disembodied or environment-centered viewpoint (Zhong and Kozhevnikov, 2016; Colombo et al., 2017; see also Klatzky, 1998). Previous neuroimaging work by Iaria and colleagues (based on samples of young adults) showed double dissociation between allocentric and egocentric/response strategies, with activation in the hippocampus corresponding with the former (Iaria et al., 2003, 2007), and activation in the caudate nucleus/striatum corresponding with the latter (Iaria et al., 2003). An alternative egocentric spatial strategy, requiring the tracking and spatial updating of movements away from a point of origin during path integration (Loomis et al., 1999; Mahmood et al., 2009; Wiener et al., 2011; Zhong and Kozhevnikov, 2016), depends on the stability of grid cell firing patterns in the entorhinal cortex of both humans (Stangl et al., 2018) and mice (Gil et al., 2018). Following the thematic emphasis on extrahippocampal functions, this review shall not discuss age differences in path integration that are contingent on entorhinal or hippocampal functions (see Mahmood et al., 2009; Wiener et al., 2011; Harris and Wolbers, 2012; Stangl et al., 2018), but on age differences in non-spatial egocentric/response strategy use.

In the cognitive aging literature, the proportion of older adults preferring an egocentric/response strategy for wayfinding has been shown to be higher than the proportion of younger adults preferring the same strategy (Bohbot et al., 2012; Rodgers et al., 2012; Wiener et al., 2013). For instance, in a virtual Y-maze that can be solved by either an egocentric/response (i.e., traversing the same route learned during training irrespective of the absolute location of the goal) or allocentric (i.e., judging the absolute location of the goal area relative to the origin regardless of the learned route) strategy, Rodgers et al. (2012) found that the great majority of older adults (83%) preferred the egocentric/response strategy whereas younger adults’ preferences were relatively evenly divided between egocentric (46%) and allocentric (54%) strategies. Similar, but less striking statistics were also reported by Bohbot et al. (2012) in a study involving a virtual radial-arm maze task. In this task, egocentric/response strategy use involved counting the number of arms from the origin to find the goal objects at the end of different radial arms, whereas allocentric strategy use involved the use of surrounding cues to judge the locations of the goal objects. Post-test surveys showed that most older adults (60.7%) reported preferring an egocentric/response strategy while younger adults’ preferences were more evenly divided between egocentric (53.7%) and allocentric (46.3%) strategies.

Older adults’ lower preference for the allocentric strategy can be traced to fMRI studies comparing both young and older subjects in navigating virtual environments; these studies generally showed that older adults have either reduced (Meulenbroek et al., 2004; Moffat et al., 2006; Konishi et al., 2013) or absent activation (Antonova et al., 2009) in the hippocampal formation. Lower allocentric strategy use among the older adults could also be attributed to smaller hippocampal volumes in older than younger adults, which in turn may undermine older adults’ accuracy in spatial learning and navigational performance (Driscoll et al., 2003; Moffat et al., 2007; Konishi and Bohbot, 2013; Yuan et al., 2014; Daugherty et al., 2015). By contrast, when searching for previously encoded goal locations in virtual environments, increased activation in the striatum (mainly the caudate nucleus) was found among older egocentric/response strategy users (Konishi et al., 2013), and when older adults were focused on processing local landmark information as compared to processing allocentric boundary information (Schuck et al., 2015). This elevated striatal activity reflected a change in information processing in the hippocampal-striatal circuitry with advancing age—with spatial learning and performance in older adults being more associated with the striatum, as well as with the prefrontal cortex (Moffat et al., 2006, 2007), than with the hippocampus (Moffat et al., 2007; Konishi et al., 2013; Schuck et al., 2015). Consequently, increased engagement of the striatum, complemented by decreased engagement of the hippocampus, may offer a credible source of support for explaining older adults’ “egocentric bias” in strategy use.

Another approach to understanding why older adults find it easier to implement egocentric/response strategies was performed by Harris and colleagues (Harris et al., 2012; Harris and Wolbers, 2014). In their studies, the researchers applied a virtual plus maze that allowed flexibility in configuring goal locations and the paths of travel. Egocentric/response strategy use involved making repetitive turning responses regardless of the whereabouts of the goal, whereas allocentric strategy use involved judging the absolute location of the goal regardless of the turning responses. Older adults attained comparable performance as the young when implementing the egocentric/response strategy but became less accurate when implementing the allocentric strategy (Harris et al., 2012). Crucially, the decline in search accuracy, as well as in the number of trials learning and implementing a specific strategy, were most prominent after the older adults were instructed to switch to allocentric strategy use from previous egocentric/response strategy use (Harris et al., 2012). This egocentric-to-allocentric strategy switching deficit further applied to finding shortcuts when navigating a larger virtual town (Harris et al., 2014). Unlike the young adults who overwhelmingly reported the consistent use of shortcuts or switching to shortcuts at some time during testing, none of the older adults did so. Instead, three-quarters of the older adults reported using shortcuts inconsistently and the remaining quarter reported the consistent use of an egocentric route strategy. A re-administration of the virtual plus maze with a smaller number of trials showed that older adults’ search accuracy declined significantly (relative to younger adults) when they were attempting allocentric-to-egocentric strategy switches, suggesting an impairment in making allocentric-to-egocentric spatial transformations could also apply to older adults.

These observed deficits in strategy switching were suggested to represent an age-related degradation of the axonal circuitry in the prefrontal cortex (Pfefferbaum et al., 2005; as cited in Harris and Wolbers, 2014), and/or an age-related dysregulation of noradrenaline production and function along the anatomical pathways linking the locus coeruleus to the prefrontal cortex (Harris et al., 2012; Harris and Wolbers, 2014). Moreover, it was speculated that an age-related reduction of the functional connectivity between the prefrontal-noradrenergic network and the hippocampus may specifically account for the egocentric-to-allocentric strategy switching deficit (Harris et al., 2012; Harris and Wolbers, 2014). As for the allocentric-to-egocentric strategy switching deficit, the researchers did not associate it with the potential degradation in prefrontal-hippocampal connectivity but instead associated it with dysfunction within the noradrenergic system linking the prefrontal cortex and the locus coeruleus (Harris and Wolbers, 2014). The locus coeruleus is strongly implicated in strategy switching in view of its tonic and phasic outputs of noradrenaline to the prefrontal cortex (Aston-Jones and Cohen, 2005). Specifically, it has been proposed that tonic locus coeruleus-noradrenergic activity facilitates disengagement from a current behavior and the selection of alternative behavior (Aston-Jones and Cohen, 2005), whereas phasic locus coeruleus-noradrenergic activity promotes the adoption of behavioral alternative(s) and the organization of functional neural networks for tackling specific tasks (Aston-Jones and Cohen, 2005; Bouret and Sara, 2008).

Worthy of extra consideration was that older adults’ difficulties with bidirectional switching between egocentric and allocentric strategies might be attributed to lower activation in the RSC (i.e., when encoding spatial information regarding the relative locations of landmarks, see Moffat et al., 2006, Table 1). This relates well to proposals of the RSC as being involved in translating between egocentric and allocentric reference frames (Maguire, 2001; Byrne et al., 2007; Vann et al., 2009; Mitchell et al., 2018), and in mediating between different spatial representations and modes of processing (Mitchell et al., 2018). Recent studies supported these proposals by showing that the RSC is crucial for: (a) judging egocentric views of target objects from rotated viewpoints that require the retrieval of allocentric spatial information (Sulpizio et al., 2016), and (b) the implementation of allocentric reference frames for integrating egocentric heading directions across separate locales (Shine et al., 2016).

Furthermore, using multi-voxel pattern analysis (MVPA), Marchette et al. (2014) showed that RSC activity patterns were similar when participants faced similar directions or imagined similar locations across four virtual museums that are geometrically similar but separated perpendicular to each other in global space. Reaction times were also faster for homologous/matching directions and locations than for non-matching directions and locations both within and across museums. Interestingly, these findings offer an alternative view of the RSC as being involved in anchoring internal spatial representations to local topographical features. However, Marchette et al. (2014) did not assess the extent to which their participants integrated local spatial representations into a global schematic map of the environment, and thus there remains the possibility (as pointed out by the authors) that the so-called “local” spatial representations of some participants could be environment-centered in nature, encompassing the spatial relationships between all landmarks imaginable. Interestingly, Mitchell et al. (2018) did not regard these findings as refuting the proposed role of the RSC in mediating between spatial reference frames and suggested that there may be RSC cells that are separately responsible for local/egocentric and global/allocentric orientation.

## Associative Learning

In addition to the challenges faced by older adults in implementing allocentric navigation strategies and switching between strategies, they also experience difficulties with the association of the correct heading directions (to goal) with landmarks or locations at which directional changes are required (Head and Isom, 2010; Liu et al., 2011; Wiener et al., 2012, 2013; Zhong and Moffat, 2016; Allison and Head, 2017; O’Malley et al., 2018). Specifically, older adults have been found to be less competent than younger adults at selecting correct headings or turns at intersections (Wiener et al., 2012, 2013; Zhong and Moffat, 2016), especially when approaching intersections from directions that are reversals of the directions encountered during route learning (Wiener et al., 2012, 2013). Notably, Wiener et al. (2013) showed that older adults’ difficulties with finding the correct heading direction when traveling the route in reverse were associated with older adults’ implementation of an associative cue strategy (i.e., “Turn left/right at”), and proposed that this strategy engaged the dorsolateral striatum.

Also noteworthy are the findings by Zhong and Moffat (2016), which showed that older adults exhibited a landmark-direction associative memory deficit at intersections, inasmuch that older adults performed poorer than younger adults at associating correct/goal-directed heading directions with varying views of landmarks at intersections but did *not* perform poorer than younger adults when tested on the recognition memory of landmarks alone. The authors speculated that this selective deficit in “binding” landmark and directional information was related to senescent changes in the fronto-hippocampal circuitry (Li et al., 2001, 2005), as characterized by degraded functional connectivity between the prefrontal cortex and the hippocampus in older adults (Grady et al., 2003). Interestingly, this proposed involvement of the fronto-hippocampal circuitry in forming landmark-direction associations parallels the relevance of the same system in making successful egocentric-to-allocentric strategy switches (Harris et al., 2012; Harris and Wolbers, 2014).

## Executive Functioning and Attention

The proposed involvement of the prefrontal cortex in both navigation-related associative learning and strategy switching necessitates a closer look at its relevance for spatial navigation. When navigating towards a specific goal location, the prefrontal cortex mediates many goal-directed processes (Spiers, 2008). Spontaneously thinking about the goal location and the best route or path to reach it have been shown to be related to increased activity in the anterior prefrontal cortex (BA 10), the dorsomedial prefrontal cortex (BA 9; in middle-aged London Taxi drivers, Spiers and Maguire, 2006, 2007), and the ventrolateral prefrontal cortex (in a college-aged sample, Carrieri et al., 2018). On the other hand, the ventromedial prefrontal cortex has been related to maintaining the intention to reach the goal in working memory and suppressing irrelevant information [as suggested by Spiers (2008), based on evidence from a patient with ventromedial prefrontal damage documented by Ciaramelli (2008)], and exerting top-down control in mediating between a hippocampus-dependent boundary processing strategy and a striatum-dependent landmark-based strategy (in a college-aged sample, Doeller et al., 2008).

When comparing young and older adults in learning the spatial layout of a virtual environment from self-directed navigation, Moffat et al. (2006) showed that older adults exhibited higher activations in the medial frontal gyrus (BA 10; see Table 1) and the anterior cingulate gyrus than younger adults, whereas younger adults exhibited higher activations than older adults in the hippocampal formation and posterior extrahippocampal areas such as the RSC, parietal cortex (i.e., inferior parietal lobule, precuneus) and angular gyrus. The authors suggested that these findings reflected a potential age-related compensatory shift in spatial memory performance toward anterior frontal systems away from medial temporal and posterior brain systems.

Notably, the authors also considered the possibility that the higher frontal activations of older adults could simply reflect age group differences in performance, since the older adults made slower virtual movements and more errors when recalling landmark locations. This alternative view relates well to other findings showing that older adults performed poorer than younger adults *even on the first trial* of a virtual Morris water maze (vMWM) when either group had no prior knowledge of the hidden platform’s location (Moffat and Resnick, 2002; Driscoll et al., 2005; Moffat et al., 2007). This suggests that older adults may possess impaired executive or strategic functions that are mediated by frontal areas at the outset of a navigational task (Moffat, 2009). Maintaining a high integrity of prefrontal gray and white matter in old age may help to attenuate or offset such executive declines, as greater volumes of lateral prefrontal gray matter and white matter have been shown to correlate positively with accurate vMWM search performance in in the first trial and onwards (Moffat et al., 2007). These findings corresponded well with additional findings showing robust negative associations between perseverative errors from the Wisconsin Card Sorting Test and vMWM search accuracy, reinforcing the proposed role of the prefrontal cortex in mediating successful navigational/search performance (Moffat et al., 2007).

In addition, recent findings by Ariel and Moffat (2018) showed that older adults attained lower metacognitive confidence judgments than younger adults in a cognitive mapping task, and that these confidence judgments mediated the relationship between age and allocentric strategy use. As the prefrontal cortex has been widely implicated as the neural basis of metacognition (see Fleming and Dolan, 2012), these findings suggest that the differential engagement of prefrontal processes in metacognition could partially account for robust age-related differences in allocentric strategy use.

Furthermore, it is important to acknowledge the effects of motor control on age-related differences in navigational performance, particularly in virtual environments. When navigating virtual environments, older adults have been observed to move more slowly when completing learning trials than younger adults (e.g., Driscoll et al., 2005; Moffat et al., 2006; Taillade et al., 2013a). This has been dealt with by using movement speed or some motion-related variables as covariates (e.g., Moffat et al., 2001, 2006; Zhong et al., 2017). While important, this only partially addresses potential qualitative effects of motor control on older adults’ navigational performance. This is because older adults’ slower movements effectively increase the delay or retention intervals across learning trials, and sets up dual tasking scenarios (e.g., attending to stimuli on screen and manipulating the control device simultaneously) for older adults that may not necessarily apply to younger adults. More specifically, prefrontal executive resources have been shown to be vital for older adults in mediating the dual task demands of visual and motor processing (Taillade et al., 2013a), as well as in supporting gait (Szturm et al., 2017). Declines in these functions may thus predispose older adults to slower movements and poorer cognitive performance in both virtual (Taillade et al., 2013a,b, 2016; Szturm et al., 2017) and real-world (Taillade et al., 2016) environments.

Together with these concerns, age-related executive declines have also been suggested to adversely affect the allocation of attentional resources (Hartmeyer et al., 2017; Merriman et al., 2018). Specifically, older adults were slower than younger adults in responding to auditory probes at intersections when simultaneously deciding on the correct heading directions to take, especially when turning movements were required (Hartmeyer et al., 2017). Older adults were also distracted by crowds of moving pedestrians and demonstrated lower accuracy in finding the correct heading directions at intersections in the presence of such crowds (Merriman et al., 2018). This susceptibility to distraction in the presence of other moving agents may have been brought about by a corresponding age-related reduction in goal-tracking eye movements or saccades (Dowiasch et al., 2015).

Overall, it is important to understand the challenges older adults experience under dual task and attention-demanding scenarios because such knowledge will invariably inform future efforts at investigating how such challenges can be managed or ameliorated.

## Summary and Future Directions

Taken together, this survey of spatial navigation studies in the cognitive aging literature suggests a shift away from hippocampal engagement toward increased engagement of extrahippocampal areas, notably the striatum and the prefrontal cortex, with advancing age. While extant neuroimaging studies have provided reliable findings showing that an increased engagement of the striatum, coupled with reduced engagement of the hippocampus, could account for older adults’ egocentric/response strategy use (Konishi et al., 2013; Schuck et al., 2015), the findings concerning age-related deficits in strategy switching (Harris et al., 2012; Harris and Wolbers, 2014), and landmark-direction associative learning (Zhong and Moffat, 2016) were behavioral in nature and speculative. It thus remains unknown as to whether dysfunctions in the fronto-hippocampal and the fronto-locus coeruleus circuits (as implicated in associative learning and strategy switching) would indeed be related to age-related declines in navigational ability. It is also possible that dysfunctions in these circuits would adversely affect navigational ability regardless of age and they may be *essential* for successful navigation. Moreover, we do not yet know whether the association of landmark and directional information is distinct from the association of other types of information (e.g., paired word associates, name-picture pairs, see Cabeza and Dennis, 2012). It thus remains possible that the brain systems related to the associations of these different pieces of information may be dissociable. Further investigations are definitely needed to clarify all these possibilities.

In addition, considering the importance of the prefrontal cortex for strategy switching, associative learning and mediating executive functions, future spatial navigation studies can take a system-oriented approach that investigate the interaction between prefrontal cortex, the hippocampus, the striatum, and/or other navigationally relevant regions (e.g., the RSC and precuneus; see, e.g., Brown et al., 2012; Sherrill et al., 2013; Chrastil et al., 2017) in both young and older adults. Most likely, this endeavor will present a broader picture of how age-related changes in prefrontal activity mediates or coordinates corresponding changes in activities in both hippocampal and extrahippocampal regions during spatial navigation.

Finally, it may also be important to clarify the significance of increased prefrontal activity in older adults during spatial navigation. Specifically, *does higher (pre)frontal activation in older adults represent an adaptive (or maladaptive) compensation mechanism among older adults?* (cf. Grady et al., 1994; Gutchess et al., 2005). To address this question, further investigations can adopt an individual differences approach when assessing older adults, as some recent studies have demonstrated that older adults with relatively high cognitive functions (O’Malley et al., 2018) or intact spatial learning ability (Zhong et al., 2017) can perform as well as younger adults in virtual navigation tasks. Consequently, the separate analysis of frontal activation profiles of older adults with differential levels of navigational performance or strategy preference shall provide greater insight into how older adults engage navigationally relevant executive processes.

## Author Contributions

JZ and SM conceived the framework for the review. Both authors conducted literature review and drafted the manuscript. Both authors edited the manuscript and finalized it for publication.

## Conflict of Interest Statement

The authors declare that the research was conducted in the absence of any commercial or financial relationships that could be construed as a potential conflict of interest. The reviewer JMW declared a past co-authorship with one of the authors SM to the handling Editor.
